# MAPK Signal Transduction Pathway Regulation: A Novel Mechanism of Rat HSC-T6 Cell Apoptosis Induced by FUZHENGHUAYU Tablet

**DOI:** 10.1155/2013/368103

**Published:** 2013-05-16

**Authors:** Qi Wang, Hongbo Du, Min Li, Yue Li, Shunai Liu, Ping Gao, Xiaoli Zhang, Jun Cheng

**Affiliations:** ^1^Institute of Infectious Diseases, Beijing Ditan Hospital, Capital Medical University, Beijing 100015, China; ^2^Beijing Key Laboratory of Emerging Infectious Diseases, Beijing 100015, China; ^3^Department of Traditional Chinese Medicine, Beijing Ditan Hospital, Capital Medical University, Beijing 100015, China; ^4^Beijing Center for Physical and Chemical Analysis, Beijing 100094, China

## Abstract

FUZHENGHUAYU Tablets have been widely used in the treatment of liver fibrosis in China. Here, we investigate the apoptotic effect of FUZHENGHUAYU Tablet in rat liver stellate cell line HSC-T6. HSC-T6 cells were incubated with control serum or drug serum from rats fed with 0.9% NaCl or FUZHENGHUAYU Tablet, respectively. Cells exposed to drug serum showed higher proportions of early and late apoptotic cells than controls. The mRNA levels of collagens I and III, TGF-*β*1 and *α*-SMA were reduced by drug serum compared to control serum. Differentially expressed mRNAs and miRNAs were analyzed by microarray and sequencing, respectively. We identified 334 differentially expressed mRNAs and also 60 GOs and two pathways related to the mRNAs. Seventy-five differentially expressed miRNAs were down-regulated by drug serum and 1963 target genes were predicted. 134 GOs up-regulated in drug serum group were linked to miRNA targets, and drug serum also regulated 43 miRNA signal transduction pathways. Protein levels were evaluated by Western blot. Drug serum down-regulated (phospho-SAPK/JNK)/(SAPK/JNK) and up-regulated phospho-p38/p38 ratios. The study showed that FUZHENGHUAYU Tablet induced apoptosis in rat HSC-T6 cells possibly in part by activating p38 and inhibiting SAPK/JNK.

## 1. Introduction

Liver fibrosis is a consequence of chronic liver disease characterized by replacement of liver tissue by fibrosis, scar tissue, and regenerative nodules, leading to loss of liver function. The condition is associated with various types of liver injury, including viral hepatitis, alcohol abuse, nonalcoholic steatohepatitis (NASH), autoimmunity, drug intoxication, and primary biliary cirrhosis. While viral hepatitis remains the leading cause of liver transplantation globally, the prevalence of non-alcoholic fatty liver disease (NAFLD) has escalated over the last decade and is increasingly being recognized as a cause of liver cirrhosis and hepatocellular carcinoma (HCC) [1, 2]. In order to prevent the development of end-stage liver diseases, it is necessary to control or reverse fibrosis. However, there is currently no high-efficient therapy method for this condition.

Liver fibrosis results from chronic damage to the liver in conjunction with the alterations in both the quantity [3] and composition of extracellular matrix (ECM) proteins [4], including collagens (I, III, and IV), fibronectin, undulin, elastin, laminin, hyaluronan, and proteoglycans. Hepatic stellate cells (HSCs) are the main ECM-producing and regulating cells in the injured liver [5]. HSCs reside in the space of Disse (perisinusoidal space) in the normal liver. Following chronic injury, HSCs transdifferentiate into myofibroblast-like cells and acquire contractile, proinflammatory, and fibrogenic properties [6, 7].

Interactions between different cellular components are thought to be involved in the disease process. A number of abnormal candidate expression genes and miRNAs have been proposed which could affect the progression of hepatic fibrosis [8]. Transforming growth factor-beta 1 (TGF-*β*1) from both paracrine and autocrine sources has been shown to be the key mediator of hepatic fibrogenesis [9], and alpha-smooth muscle actin (*α*-SMA) is an indicator of stellate cell activation [10]. MiR-29b, -199a, -199a*, -200a, and -200b have been reported related to the process of liver fibrosis [11, 12]. It has also been proposed that miRNA changes regulated by negative feedback loops between miRNAs and their downstream genes might play an important role in the steady-state regulation [13]. So, detailed mechanism of liver fibrosis involved in genes and miRNAs still needs to be further clarified. In addition, in mammalian cells, microRNAs (miRNAs) usually bind to complementary sites in the 3′ untranslated region (3′-UTR) of specific target genes, resulting in reduced gene translation. Since miRNAs do not generally affect mRNA level, gene expression profiling is not suitable for exploring the target signaling pathways of miRNA. Instead, miRNA-mRNA interaction network analysis is widely used to the medical researches [14]. This is also consistent with the view that the focus of cataloging a “parts list” of genes and proteins should be changed to a strategy of mapping the network of interactions between them [15].

Many kinds of traditional Chinese medicines (TCMs) have been shown to have antifibrotic properties. These include Ganoderma lucidum (Ling Zhi) [16], Sinisan [17] and Shugan-Huayu powder [18]. TCM 319, also known as FUZHENGHUAYU Tablet, is a compound containing six Chinese herbs, including Radix Salviae Miltiorrhizae, Fermentation Mycelium Powder, Semen Persicae, Fructus Schisandrae, Chinensis Pollen pini, and Gynostemma pentaphyllammak [19]. Previous studies have shown that FUZHENGHUAYU has antifibrotic effects in rats [19–25]. But there are no reports of the effects of FUZHENGHUAYU on HSC apoptosis, and little is known about the role played by miRNA and mRNA related mechanisms with respect to the effects of FUZHENGHUAYU Tablet. In this study, we used the estalished methods and other technologies to investigate the molecular mechanisms of FUZHENGHUAYU Tablet in liver fibrosis.

## 2. Materials and Methods

### 2.1. Drug Serum and Control Serum Preparation

Twelve male Wistar rats, SPF grade, weighing 300–350 g, were divided equally into control and drug groups. FUZHENGHUAYU Tablet was suspended in distilled water at concentration of 0.04 g/mL. The drug group received FUZHENGHUAYU Tablet dilution at a dose of 2 mL/100 g·wt, twice daily for 3 days. Two hours after the last dose of dilution, serum was collected from the inferior vena cava and inactivated at 56°C for 30 minutes. Serum samples were stored at −70°C until further processing. The control group rats were treated with 0.9% NaCl and were subjected to the same procedure [22]. This study was performed according to the international, national, and institutional rules considering animal experiments. In present study, drug and control serums were supplied by Dr. Chenghai Liu, Institute of Liver Disease, Shanghai University of Traditional Chinese Medicine, China.

### 2.2. Cell Culture, Grouping, and Treatment

Rat HSC-T6 cells from our laboratory were cultured in Dulbecco's modified Eagle's medium (DMEM; Gibco) containing 10% fetal bovine serum (FBS; Gibco), 100 U/mL penicillin, and 100 *μ*g/mL streptomycin (Gibco) in a humidified chamber at 37°C in 5% CO_2_. For the control and drug groups, control and drug serums were used instead of FBS, respectively. The cells were cultured for up to 24 h for RNA and protein extraction and for 72 h for apoptosis analysis. Actinomycin D (2 *µ*L/mL; Sigma) was added into rat HSC-T6 cells exposed to 10% FBS after 12 h as a positive control for the analysis of apoptosis.

### 2.3. Apoptosis Analysis

Rat HSC-T6 cells treated with drug serum or control serum were washed twice in cold PBS and harvested by exposure to trypsin-EDTA solution. The harvested cells were centrifuged, washed with complete media, and then suspended in Annexin V binding buffer. Apoptosis was assessed using an Annexin V/7-AAD apoptosis kit according to the manufacturer's protocol (Biolegend). Fluorescein isothiocyanate (FITC)- Annexin V, and 7-AAD were added to the cell suspension. The cells were incubated for 30 min at room temperature in the dark and analyzed using a BD FACSCalibur flow cytometer. After fluorescence activated cell sorting (FACS), the percentage of apoptotic cells was assessed using ModFit software.

### 2.4. Total RNA Extraction

Total RNA was extracted using an EZNA total RNA kit (Omega), according to the manufacturer's instructions. For gene expression microassays and miRNA sequeneing, total RNA was extracted using mirVana miRNA isolation kit (Ambion) and checked for RIN number to inspect RNA integration by an Agilent Bioanalyzer 2100 (Agilent technologies). Only samples with RNA integrity > 7 and with 28S/18S ≥ 0.7 were used in the further analysis. RNA samples were stored at −80°C until further processing.

### 2.5. Real-Time PCR

An SYBR ExScript RT-PCR Kit (TAKARA) and Power SYBR Green PCR Master Mix (ABI) were used for real-time PCR. The primers of rat collagen I, collagen III, TGF-*β*1, *α*-SMA, and *β*-actin are listed in Table 1 [26]. Beta-actin was used as an endogenous control. Reactions for each sample were performed in triplicate with equal amounts of template cDNA, using the ABI Prism 7500 Sequence Detection System. Real-time PCR conditions were as follows: 95°C for 10 min, followed by 40 cycles at 95°C for 15 s and 60°C for 1 min. Fold induction values were calculated using the 2^ΔΔCt^ method according to the manufacturer's instructions.

### 2.6. Microarray Hybridization and Data Analysis

Total RNA (100 ng) was amplified, labeled, and purified by using GeneChip 3′IVT Express Kit (Affymetrix) to obtain biotin labeled cRNA. Labeling and hybridization were performed at Shanghai Biochip Company according to the protocols in the Affymetrix Rat 230 2.0 microarray system. Raw data were normalized using the MAS 5.0 algorithm (Gene Spring Software 11.0; Agilent technologies). 

After feature extraction (Feature Extraction software), log 2 ratios, representing the ratio of Cy5-processed signal to Cy3-processed signal, were calculated and converted to fold changes. Genes with a log 2 ratio >1 (>2-fold increase) were considered to be upregulated, and those with <−1 (>2-fold decrease) were considered to be downregulated.

### 2.7. MicroRNA Profile Sequencing and Target Prediction

The small RNA libraries were constructed following the manufacturer's instructions for the Small RNA Sample Prep Kit (Illumina). The small RNAs were ligated with adapters followed by reverse transcription and amplification. The PCR products derived from 22 nt and 30 nt small RNA fragments were purified from 6% Novex TBE PAGE Gel. Purified miRNAs were sequenced on the Illumina Genome Analyzer for 36 cycles. Sequencing was performed at Shanghai Biotechnology Corporation. MiRNAs genes with fold change ≥2 or ≤0.5, *P* value ≤ 0.05, and FDR ≤ 0.05 were considered to be up-regulated, and miRNAs genes with fold change ≤0.5, *P* value ≤ 0.05, and FDR ≤ 0.05 were considered to be down-regulated. Targetscan (http://www.targetscan.org/) was used for miRNA target prediction. 

### 2.8. Gene Ontology (GO) Category and Pathway Analysis

DAVID gene database annotation (DAVID Bioinformatics Resources 6.7) was used to interpret the biological effect of mRNAs and target genes of miRNAs. The categorization of the biological process GO of the difference expression genes and target genes was analyzed using the Gene Ontology project (http://www.geneontology.org/) which is the key functional classification of the National Center for Biotechnology Information (NCBI). The KEGG genome database was used to identify significant differential genes pathways. In view of the large differences in enrichment numbers, different *P*-values (≤0.05, ≤0.01, and ≤0.001) were used as a threshold to select significant gene ontology (GO) categories and KEGG pathways, each representing significant differences.

### 2.9. Western Blot Analysis

Cultured cells were lysed using Proteo JET Mammalian Cell Lysis Reagent (Fermentas) containing a cocktail of proteinase inhibitors (Roche) and phosphatase inhibitor cocktail (Pierce). The debris was discarded and the supernatant containing total proteins was quantified using a BCA kit. The proteins were run on 10% SDS-PAGE and transferred onto PVDF membranes (Millipore, Bedford). After blocking in 5% nonfat milk, the membranes were probed with rabbit monoclonal antibodies against rat p44/42 MAP kinase (137F5) (CST), SAPK/JNK (56G8) (CST), p38 MAP kinase (CST), phospho-p44/42 MAPK (Thr202/Tyr204) (CST), phospho-p38 MAPK (Thr180/Tyr182) (CST), phospho-SAPK/JNK (Thr183/Tyr185) (CST), and mouse monoclonal antibody against rat *β*-actin (Santa Cruz) as primary antibodies at 1 : 1000 dilution. Goat anti-rabbit or goat anti-mouse IgG labeled with HRP (1 : 2000) were used as secondary antibodies. Immunoreactive signals were detected using an Enhanced Chemiluminescence kit (Amersham Pharmacia Biotech) through an ECL system. The results were quantified using the Image J 1.43 software (National Institutes of Health, Bethesda, MD) after densitometric scanning of the films.

### 2.10. Statistical Analysis

Data were expressed as means ± SD. Differences between experimental groups were assessed using the two-tailed *t*-test. Statistical significance was defined as *P < 0.05 and **P < 0.01.

## 3. Results 

### 3.1. FUZHENGHUAYU Tablet Induces Apoptosis in Rat HSC-T6 Cells

**Figure 1:**
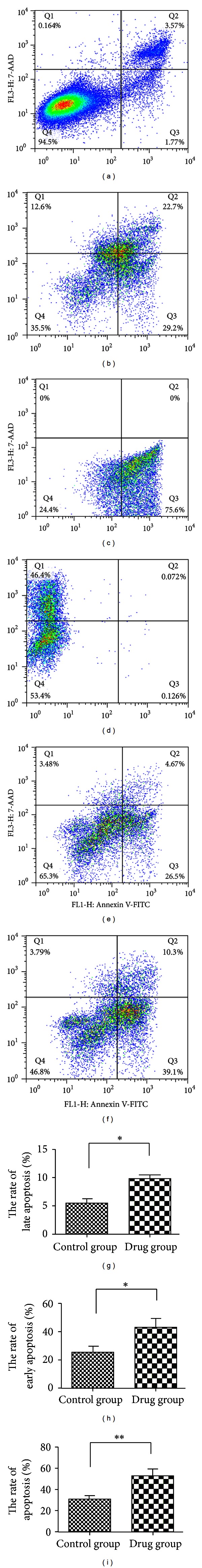
FUZHENGHUAYU Tablet induced apoptosis in rat HSC-T6 cells. Cultured rat HSC-T6 cells were divided into control and drug groups. The control group was incubated with DMEM medium containing 10% control serum, and the drug group was cultured with DMEM medium supplemented 10% drug serum for up to 72 h. Apoptosis was assessed using an Annexin V/7-AAD Apoptosis kit and analyzed using a BD FACS Calibur flow cytometer. (a) HSC-T6 cells exposed to 10% FBS and stained with Annexin V(+)/7-AAD(+); (b) HSC-T6 exposed to 10% FBS + 2 *μ*L/mL actinomycin D and stained with Annexin V(+)/7-AAD(+); (c) HSC-T6 cells exposed to 10% FBS + 2 *μ*L/mL actinomycin D and stained with Annexin V(+)/7-AAD(−); (d) HSC-T6 cells exposed to 10% FBS + 2 *μ*L/mL actinomycin D and stained with Annexin V(−)/7-AAD(+); (e) HSC-T6 cells exposed to 10% control serum and stained with Annexin V(+)/7-AAD(+). (f) HSC-T6 cells exposed to with 10% drug serum and stained with Annexin V(+)/7-AAD(+); (g) the late apoptotic cells in the drug (10%  ± 1%) and control groups (5%  ± 1%) (*n* = 3, *P < 0.05); (h) the early apoptotic cells in the drug (43%  ± 6%) and control groups (26%  ± 4%) (*n* = 3, *P < 0.05); (i) total apoptotic cells in the drug (53%  ± 6%) and control groups (31%  ± 4%) (*n* = 3, **P < 0.01).

In order to analyze whether FUZHENGHUAYU Tablet could induce apoptosis in rat HSC-T6 cells, we treated rat HSC-T6 cells with control or drug serum, respectively. Cells were stained with Annexin V-FITC/7-AAD and gated into lower right (LR) and upper right (UR) quadrants. Cells in LR and UR represented early (Annexin V(+)/7-AAD(−)) and late apoptotic (Annexin V(+)/7-AAD(+)) cells, respectively. Cells in lower left (LL) quadrants were considered to be alive and those in the upper left (UL) quadrants were considered to be necrotic. The extent of apoptosis was expressed as the sum total of the percentages in LR and UR quadrants. The apoptotic rates are showed in Figures 1(a) to 1(f). Cells exposed to drug serum showed more late apoptotic cells (10%  ± 1%) than control serum (5%  ± 1%) (*n* = 3, *P* < 0.05). They also contained a higher proportion of early apoptotic cells (43%  ± 6%) than the controls (26% ± 4%) (*n* = 3, *P* < 0.05). The total apoptotic cells in the drug group and control group were 53%  ± 6% and 31%  ± 4%, respectively (*n* = 3, *P* < 0.01).

### 3.2. FUZHENGHUAYU Tablet Decreased mRNA Levels of Collagen I, Collagen III, TGF-*β*1, and *α*-SMA

**Figure 2:**
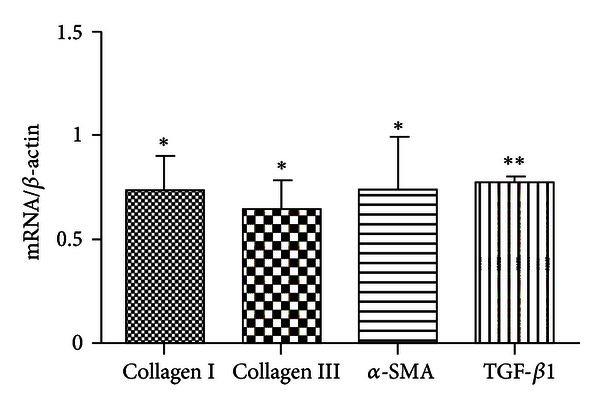
FUZHENGHUAYU Tablet serum decreased the mRNA levels of collagen I, collagen III, TGF-*β*1, and *α*-SMA. Data are expressed as mean ± SD. After incubation with drug serum or control serum for up to 24 h, the mRNA levels of collagen I, collagen III, TGF-*β*1, and *α*-SMA were significantly down-regulated (*n* = 3, *P < 0.05, **P < 0.01), being 0.73-, 0.64-, 0.74-, and 0.78-fold lower than the control group, respectively.

The levels of collagen I, collagen III, TGF-*β*1, and *α*-SMA were potential markers of the antifibrosis efficacy of FUZHENGHUYU Tablet. We found that mRNA levels of collagen I, collagen III, TGF-*β*1, and *α*-SMA was significantly down-regulated after exposure of rat HSC-T6 cells to drug serum for up to 24 h. The degree of expression was 0.73-, 0.64-, 0.74-, and 0.78-fold lower than in the control serum group (Figure 2). These findings suggest that apoptosis may be the mechanism of the anti-fibrotic activity of FUZHENGHUAYU Tablet.

### 3.3. Differentially Expressed mRNA in Control Serum and FUZHENGHUAYU Tablet Serum Groups

Microarray profile analyses of cellular mRNAs in rat HSC-T6 cells identified 334 mRNAs that were differentially expressed between the drug serum group and control serum group; 199 mRNAs were up-regulated and the remainder were down-regulated.

### 3.4. Differentially Expressed miRNAs in FUZHENGHUAYU Tablet Serum and Control Serum Groups Target Gene Prediction

MicroRNA profile sequencing identified 75 differentially expressed miRNAs, which were down-regulated in drug serum group in comparison with the control serum group. Targetscan (http://www.targetscan.org/) prediction of miRNAs identified 1963 potential target genes.

### 3.5. Bioinformatics Interpretation Revealed the GOs and Signaling Pathways Regulated by Differentially Expressed mRNAs and miRNAs

In order to gain insights into the function of miRNAs and mRNAs, GO term and KEGG pathway annotation were applied. 

**Figure 3:**
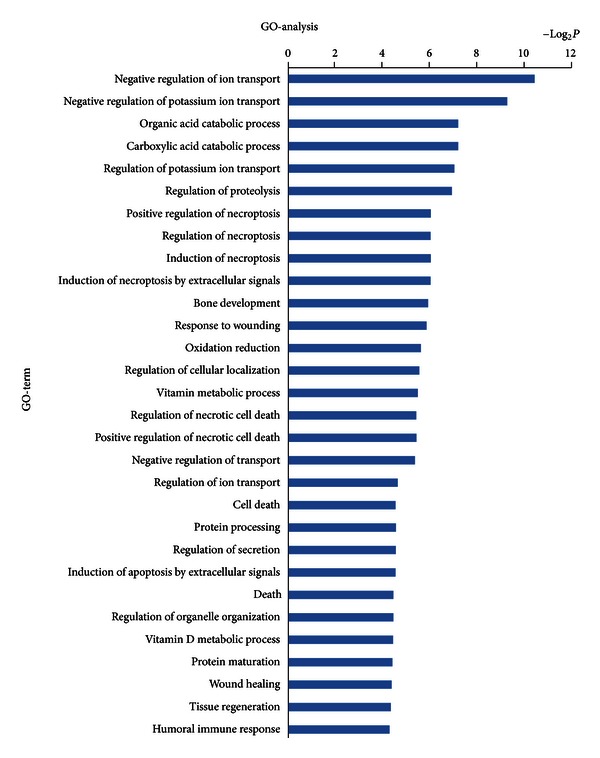
Bioinformatics interpretation revealed the GOs regulated by up-regulated expressed mRNAs. Genes with up-regulated expression in the drug and control groups were analyzed by GO. *P* values ≤ 0.05 were used as a threshold to select significant GO categories.

**Figure 4:**
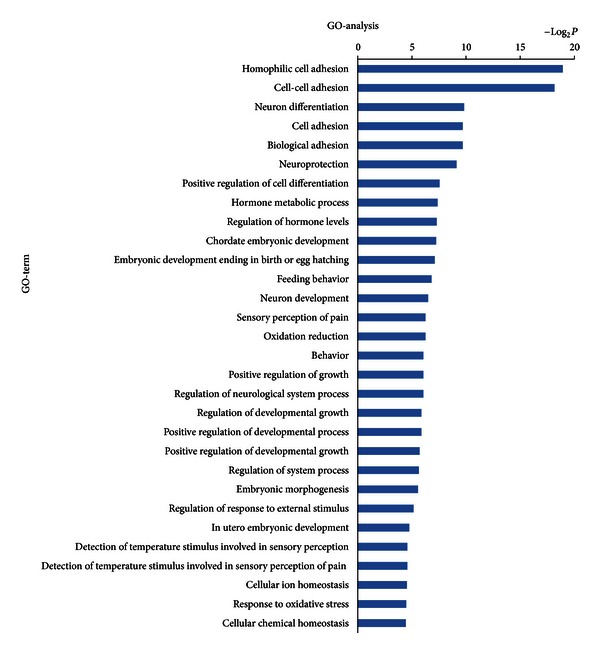
Bioinformatics interpretation revealed the GOs regulated by down-regulated expressed mRNAs. Genes with down-regulated expression in the drug and control groups were analyzed by GO. *P* values ≤ 0.05 were used as a threshold to select significant GO categories.

**Figure 5:**
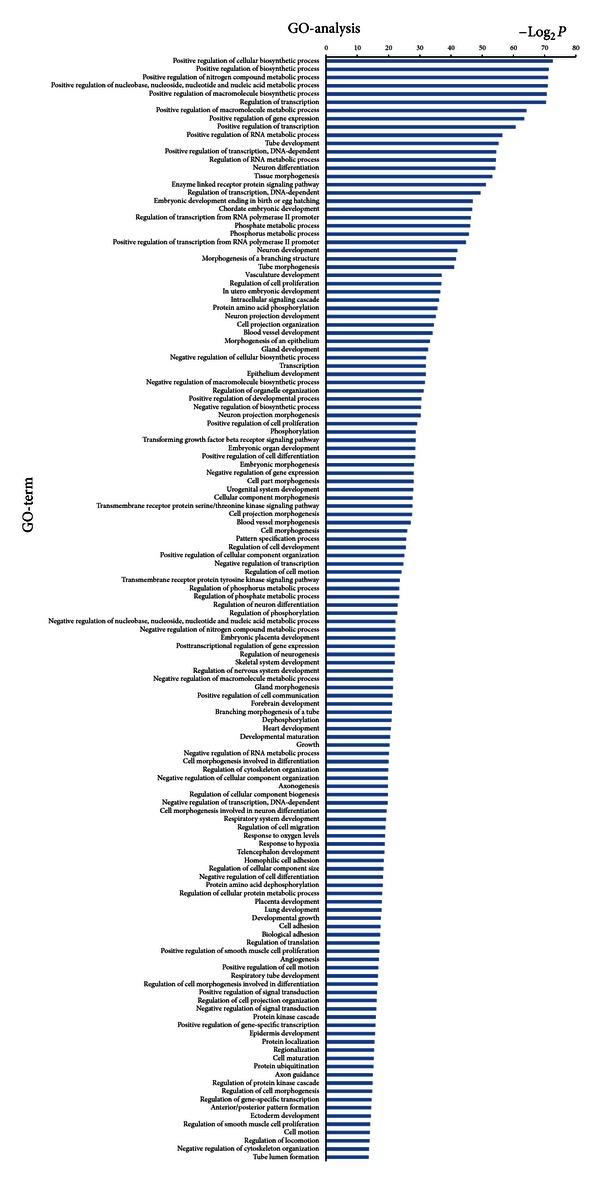
Bioinformatics interpretation revealed the GOs regulated by differentially expressed miRNAs target genes. Genes with significant expression difference in the drug and control groups were analyzed by GO. *P* values ≤ 0.001 and FDR ≤ 0.05 were used as a threshold to select significant GO categories.

We have identified 30 up-regulated GOs (Figure 3) and 30 down-regulated GOs (Figure 4) on the differentially expressed mRNAs. These genes were involved in ion transport, necroptosis, cell death, metabolic processes, cell development, differentiation, and adhesion. This form of analysis also identified 134 upregulated GOs by differentially expressed miRNAs target genes (Figure 5), which could be categorized as cell processes, gene expression, cell development, morphogenesis, signaling pathways, cell organization, proliferation, adhesion, and so on. Among all the differentially regulated GOs, those involved in cell development, adhesion, growth, necroptosis, and transport appeared to predominate.

**Figure 6:**
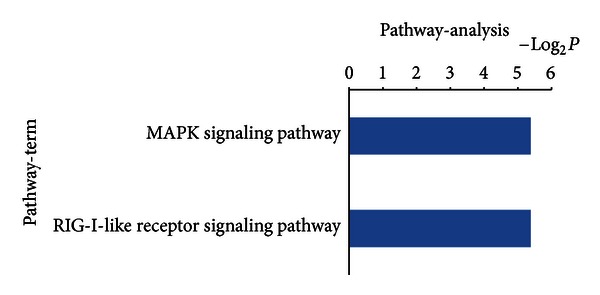
Bioinformatics interpretation revealed the KEGG genomes regulated by differentially expressed mRNAs. Genes with significant expression differences in the drug and control groups were analyzed by KEGG. *P*-values ≤ 0.05 were used as a threshold to select significant KEGG analysis.

**Figure 7:**
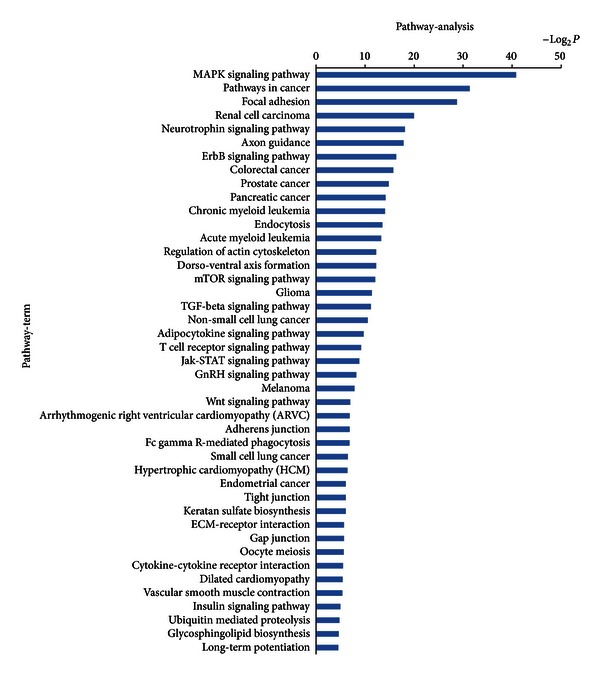
Bioinformatics interpretation revealed the KEGG genomes regulated by mRNAs of differentially expressed miRNAs. Genes with significant difference in the drug and control groups were analyzed by KEGG. *P*-values ≤ 0.01 were used as a threshold to select significant KEGG analysis.

Additional functional analysis of mRNAs using KEGG analysis identified the two signal transduction pathways regulated by drug serum. These were mitogen-activated protein kinases (MAPK) and RIG-1 like receptors (Figure 6). In addition, 43 signal transduction miRNA targets were found to be regulated by drug serum. These included MAPK and various cancer signals, as shown in Figure 7. 

Enrichment ranking of signaling pathways indicated that MAPK signal transduction pathway was the most prominent. As we know, MAPK signal transduction pathways are involved in a series of important biological effects, such as inflammation, migration, apoptosis, growth, development, and differentiation [27, 28]. 

The apoptosis results, previous reports, and the results of GO and KEGG analysis all suggested that MAPK signal transduction pathway might be regulated by drug serum, and this also might be related to the induction of apoptosis in rat HSC-T6 cells.

### 3.6. MAPK Signal Transduction Pathway Was Regulated by FUZHENGHUAYU Tablet

Mammals express at least four distinctly regulated groups of MAPKs, extracellular signal-related kinases (ERK)-1/2, Jun amino-terminal kinases (JNK1/2/3), p38, and ERK5. MAPKs belong to a large family of serine/threonine protein kinases. They not only activate/inactivate other proteins but are themselves activated/inactivated by other proteins through phosphorylation/dephosphorylation modification [29].

**Figure 8:**
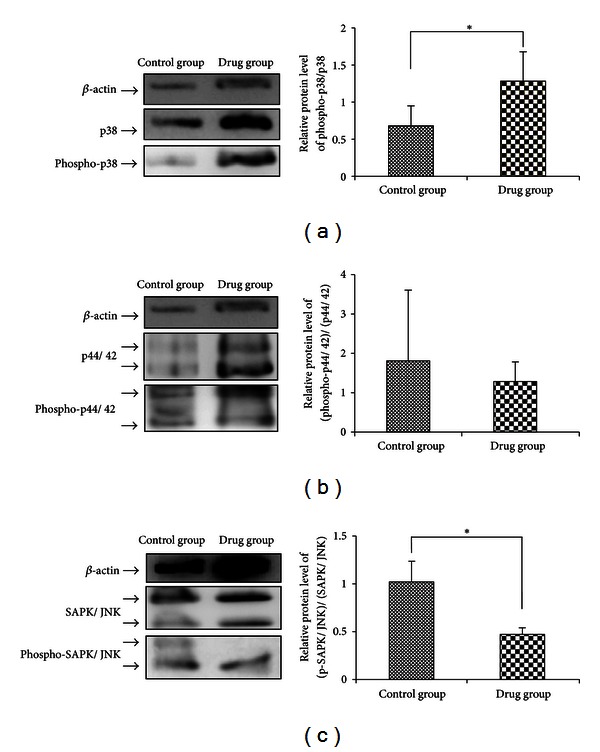
The MAPK signal transduction pathway is regulated by FUZHENGHUAYU Tablet. Protein levels of rat p44/42 MAP kinase (137F5), SAPK/JNK (56G8), p38 MAP kinase, phospho-p44/42 MAPK (Thr202/Tyr204), phospho-p38 MAPK (Thr180/Tyr182), and phospho-SAPK/JNK (Thr183/Tyr185) were detected by Western blot.

In order to analyze the hypothesis, the protein levels of rat p44/42, SAPK/JNK, p38, phospho-p44/42, phospho-p38, and phospho-SAPK/JNK were detected by Western blot. The results indicated that the ratio of (phospho-SAPK/JNK)/(SAPK/JNK) was significantly down-regulated and that the ratio of phospho-p38/p38 was significantly up-regulated by drug serum (*n* = 3, *P* < 0.05) (Figure 8). There was no significant change in the (phospho-p44/42)/(p44/42) ratio. These results suggest that changes in SAPK/JNK and p38 in response to drug serum might be related to its apoptotic effects in rat HSC-T6 cells.

## 4. Discussion

Traditional Chinese medicine (TCM) uses a holistic approach taking the human body as a self-controlled system network. The goals of biologically the based medicine partially overlap the principles of TCM. Bioinformatic and systems biology, therefore, represent an important link between TCM and Western medicine [30]. Bioinformatics and system biology have previously been used to identify disease-related genes or functional modules and to recognize redundant, adaptable, and system mechanisms in diseases [31, 32]. The previous reports have supplied us with perfect examples in network pharmacology study. 

Liver fibrosis results from the activation of HSCs as part of the wound-healing response to chronic liver injury [33]. It is known that HSCs undergo a transition from a quiescent to an activated phenotype following liver tissue damage [34]. The generation and proliferation of *α*-SMA positive myofibroblasts from the periportal and perisinusoidal areas also play a central in the fibrotic process. Previous studies have shown that FUZHENGHUAYU is able to normalize ALT and AST levels in patients with chronic hepatitis B and to some extent reverse the development of liver fibrosis [20, 21]. Another study indicated that FUZHENGHUAYU decoction prevents the autocrine activation of HSCs, possibly by inhibiting secretion of VEGF [22]. A similar study proposed that the anti-fibrotic effects of FUZHENGHUAYU may also be associated with inhibition of liver collagen production. It has also been reported that FUZHENGHUAYU extracts attenuate hepatic fibrosis induced by CCl4 in rats [19]. The same study showed that the anti-fibrotic effect of FUZHENGHUAYU was associated with down-regulation of mRNA expression of PDGF-B and PDGF-R*β* and with reduced protein expression of TGF-*β*1 [23]. In addition, spontaneous or targeted apoptosis of HSC has been shown to be associated with regression of liver fibrosis in animal models [24, 25].

 In the present study we showed for the first time that FUZHENGHUAYU Tablet was able to induce apoptosis in rat HSC-T6 cells. In order to confirm the relationship between the apoptotic and anti-fibrotic effects, we analyzed the mRNA levels of collagen I, collagen III, TGF-*β*1, and *α*-SMA. Our results showed a significant down-regulation in each case.

The miRNAs and their target genes have emerged as key regulators of diverse biological processes, including cancer [35], development [36], cell growth, apoptosis [37], and immune responses [38]. It is known that miRNAs and mRNAs both play essential roles in apoptosis, differentiation, proliferation, and migration in HSCs [8] and also are involved in the process of liver fibrosis [39]. A previous study suggested that miR-29 regulates liver fibrosis and together with TGF-*β* and nuclear factor-*κ*B forms part of a signaling nexus in HSCs [40]. It has also been shown that hepatic levels of miR-29 are significantly increased in mice with CCl4 induced liver damage and also in the livers of patients with advanced fibrosis. By contrast, miR-29b appears to act as a beneficial factor that protects against liver fibrosis by suppressing the activation of HSCs [11]. It has been suggested that upregulation of miR-199a, -199a*, -200a, and -200b s triggers the process of liver fibrosis [12]. Specifically, it has been suggested that miR-16 has the potential to inhibit HSC proliferation and induce apoptosis by inducing Bcl-2 while concurrently reducing cyclin D1 levels [37]. Other evidence suggests that overexpression of miR-181b increases the growth of HSCs by directly targeting p27 [39].

Microarray techniques are increasingly being used as research tools in chemistry and life sciences. The sequencing of the human genome, together with the development of high-throughput technologies, affords a unique opportunity for future research [41]. Microarray and high-throught sequencing are both based on computational biology and bioinformatics, and both are appropriate for mRNA and miRNA research. In this study, we identified 334 differentially expressed mRNAs. Sequencing identified 75 miRNAs with down-regulated expression in the drug serum group. Online database analysis identified 60 differently regulated GOs from mRNAs and 134 regulated GOs from miRNAs targets that were principally involved in cell development, adhesion, growth, necroptosis, and transport processes. Functional analysis of mRNAs by KEGG revealed that drug serum regulated two signal transduction pathways, involving MAPK and RIG-1 like receptors. In addition, drug serum regulated 43 miRNA signal transduction pathways, principally the MAPK pathway and to a lesser extent pathways involved in cancer signals. These findings suggested that drug serum has wide ranging effects on rat HSC-T6 cells, which resulted from differential expression of miRNAs and mRNAs. They also suggested that MAPK signal transduction might be involved in these complex processes. The number of predicted target genes was much higher than the number of differently expressed mRNAs, reflecting differences in the numbers of enriched pathways.

It is possible that liver fibrogenesis is regulated by intracellular signaling pathways, involved in apoptosis, proliferation, migration, or inflammation. Mammals express at least four distinctly regulated groups of MAPKs: ERK-1/2, JNK1/2/3, p38 proteins (p38alpha/beta/gamma/delta), and ERK5. The MAPK cascade is known to be involved in various cellular functions, including cell proliferation, apoptosis, differentiation, and migration. MAPKs have also been shown to modulate major fibrogenic actions of HSCs, but different members of the group have different effects. A previous study showed that ERK stimulation in experimentally induced liver injury mediated the proliferation and migration of HSCs [42]. Other reports have shown that p38-MAPK and caspase-3 both mediate superoxide-induced apoptosis in rat HSCs [43]. Another study showed that TAK1/JNK inhibition decreased HSC proliferation, whereas p38 inhibition increased the rate of HSC proliferation, independently of its activation status [34]. The same study showed that JNK inhibition increased and p38 inhibition decreased collagen alpha 1 (I) mRNA levels. Additional evidence indicates that c-JNK regulates hepatocyte apoptosis as well as regulating the secretion of inflammatory cytokines by cultured HSCs [44, 45]. Indole-3 carbinol (I3C) is known to inhibit the proliferation of HSC by blocking the NADPH oxidase/reactive oxygen species/p38 MAPK signal pathway [46]. A recent study has shown that p38 may play an important role in the regulation of HSC self-renewal in vitro. Based on this finding, it was suggested that inhibition of p38 activation with a small molecule inhibitor might represent a novel approach to promote ex vivo expansion of HSCs [47]. In our study, drug serum down-regulated the levels of phospho-SAPK/JNK and up-regulated phospho-p38. There was no change in phospho-p44/42. These results suggest that the changes in phospho-SAPK/JNK and phospho-p38 might in part explain the apoptotic induction effects of TMC 319 in rat HSC-T6 cells.

## 5. Conclusions

Taken together our results indicate that FUZHENGHUAYU Tablet increases apoptosis of rat HSC-T6 cells by activating p38 and inhibiting SAPK/JNK. These effects may in part explain the mechanism by which FUZHENGHUAYU Tablet protects against liver fibrosis.

## Figures and Tables

**Table 1:** Sequences of primers used for real-time PCR.

Gene	Primer sequence (5′-3′)
Collagen I	Sense: TCCTGGCAATCGTGGTTCAA
	Anti-sense: ACCAGCTGGGCCAACATTTC
Collagen III	Sense: GGTCCTGCAGGTAACAGTGGTTC
	Anti-sense: TGCTCCAGTTAGCCCTGCAA
TGF-*β*1	Sense: TGCGCCTGCAGAGATTCAAG
	Anti-sense: AGGTAACGCCAGGAATTGTTGCTA
*α*-SMA	Sense: AGCCAGTCGCCATCAGGAAC
	Anti-sense: CCGGAGCCATTGTCACACAC
*β*-actin	Sense: GGAGATTACTGCCCTGGCTCCTA
	Anti-sense: GACTCATCGTACTCCTGCTTGCTG

## Abbreviations

TCM: Traditional Chinese Medicine
HSC: Hepatic stellate cell
HCC: Hepatocellular carcinoma
NASH: Non-alcoholic steatohepatitis
NAFLD: Non-alcoholic fatty liver disease
ECM: Extracellular matrix
CCl4: Carbon tetrachloride
MMP: Matrix metalloproteinase
TIMP: Tissue inhibitor of metalloproteinase
ALT: Alanine aminotransferase
AST: Aspartate aminotransferase
*α*-SMA: Smooth muscle alpha actin
*β*-actin: Beta-actin
TGF-*β*: Transforming growth factor-beta
PDGF: Platelet-derived growth factor
PDGF-R*β*: Platelet-derived growth factor receptor beta
GO: Gene ontology
miRNA: microRNA
MAPK: Mitogen-activated protein kinase
ERK: Extracellular signal regulated kinase
p38 MAP kinase: p38 mitogen-activated protein kinase
JNK: Jun N-terminal kinase
SAPK: Stress-activated protein kinases.
